# Analysis of an Ambulatory Care Pathway for Patients With COVID-19 Utilising Remote Pulse Oximetry at a London District General Hospital

**DOI:** 10.7759/cureus.12979

**Published:** 2021-01-29

**Authors:** Jonathon Kyriakides, Aria Khani, Reginald Coleman, Charlotte Kelly

**Affiliations:** 1 Acute Medicine, Barnet Hospital, Royal Free London NHS Foundation Trust, London, GBR

**Keywords:** covid-19, ambulatory care, emergency department, pulse oximetry, hypoxaemia, discharge

## Abstract

The identification of coronavirus disease 2019 (COVID-19) patients with oxygen saturations between 90-94% who can be safely discharged from the emergency department (ED) is challenging due to the difficulty of community monitoring. A pathway consisting of home pulse oximetry with telephone follow-up was devised and implemented at a London District General Hospital to facilitate the safe discharge of these patients from the ED. Twenty patients with confirmed or suspected COVID-19 with oxygen saturations between 90%-94% were discharged on this novel ambulatory care pathway. Eighty-five percent of patients successfully avoided hospitalisation, whilst 15% were re-assessed and subsequently admitted to hospital. Home pulse oximetry monitoring was utilised to aid discharge from the ED and therefore prevent hospital admission. Telephone follow-up identified patients requiring further assessment. This study demonstrates the potential for safe ambulation of a subgroup of patients with COVID-19.

## Introduction

Coronavirus disease 2019 (COVID-19) describes the respiratory disease caused by the novel severe acute respiratory syndrome coronavirus 2 (SARS-CoV-2). In a proportion of patients, the virus causes diffuse alveolar damage and interstitial thickening, which compromises gas exchange [1]. Affected individuals have been noted to be paradoxically calm in the clinical setting; this phenomenon, termed ‘silent hypoxia’, has also been observed in experimental data [2]. Decompensation in COVID-19 patients can occur suddenly and without an obvious warning. It is therefore pertinent to have a system whereby this deterioration can be identified quickly.

In otherwise healthy individuals, oxygen saturation (SaO2) below 90% correlate with hypoxaemia [3]. Prior to the COVID-19 pandemic, patients presenting to the emergency department (ED) with an SaO2 below 94% were typically admitted for monitoring or for supplemental oxygen. During the pandemic, there was a drive into investigation of satisfactory oxygen saturation levels, acknowledging the limited hospital resources of both inpatient beds and supplemental oxygen. However, identification of patients with an SaO2 of 90-94% who could be safely discharged from the ED was difficult due to uncertainties surrounding the trajectory of the COVID-19 disease course. The infectivity of COVID-19 also limits follow-up options. Therefore, a pathway consisting of home pulse oximetry monitoring with telephone follow-up was created to facilitate safe discharge. Underpinning this pathway were the aims to reduce burden on the National Health Service (NHS) at a time of unprecedented demand and to provide safe follow-up. The primary objective was to utilise home pulse oximetry in order to prevent hospital admission. The secondary objective was to identify those needing further care or investigation.

## Materials and methods

An ambulatory emergency care pathway was devised and implemented at Barnet Hospital, Royal Free London NHS Foundation Trust, in April 2020. 

Adult patients suitable for discharge from the ED on this ambulatory emergency care pathway were selected using inclusion criteria (Table 1). To facilitate decision-making, clinicians were advised to consider admission if certain clinical features were met (Table 1). These clinical features were based upon best available evidence at the time of implementation and were also in line with consensus across the Trust. These were not defined as exclusion criteria and the final decision to discharge was made by the responsible clinician. Appropriate patients were referred to the medical registrar on-call and an ambulatory emergency care referral was made. Medical registrars were given remote training on the pathway via an email.

**Table 1:** Inclusion criteria for the pathway SaO2: oxygen saturation

Inclusion criteria for the pathway	Clinical features where admission should be considered
COVID-19 viral swab positive or high clinical suspicion	SaO_2_ <90%
SaO­_2_ of 90-94% on room air at rest	Respiratory rate >24/min
Ability to use the pulse oximeter correctly	C-reactive protein (CRP) >100mg/L
Discharge decision made by a registrar or consultant	>10% pulmonary infiltrates on chest radiograph (estimated)
	Elevated troponin
	Significant diagnosed co-morbidity (including but not limited to: chronic lung disease, ischaemic heart disease)
	Representation to healthcare

A patient information leaflet was given to patients on discharge from the ED. Patients were asked to measure their home oxygen saturations at rest, three times per day (0900hrs, 1300hrs and 1800hrs) for up to seven days. 

Patients were followed up via telephone consultation on days two, five, and seven by a clinician in the ambulatory care department. A script (which involved questions regarding pulse oximetry recordings and symptom progression) was utilised by clinicians to ensure consistency. During telephone consultations, a decision to arrange re-assessment in the ED, further telephone follow-up, or discharge from the pathway was made based on the assessment. Patients were advised to return to the ED based on standard safety netting information or if oxygen saturations measured below 90% at rest on two or more occasions within a 24-hour period. In situations of clinical ambiguity, the final decision was made by the lead consultant for ambulatory emergency care. 

Data collection occurred alongside the implementation of this ambulatory care pathway between April and May 2020. Patient demographics and medical background, in addition to investigation results and patient outcomes, were analysed. 

## Results

Between April and May 2020, 21 patients were discharged from the ED on this ambulatory care pathway with a pulse oximeter. One patient was later found to have an alternative diagnosis and was therefore not included in the results, despite still avoiding hospital admission.

Of the 20 patients included in this study, 13 were male and seven were female. The cohort had a mean age of 53. Seven patients did not have any medical co-morbidities, whilst the remainder had at least one co-morbidity. The most common co-morbidities were asthma, hypertension, and type 2 diabetes mellitus.

**Figure 1:**
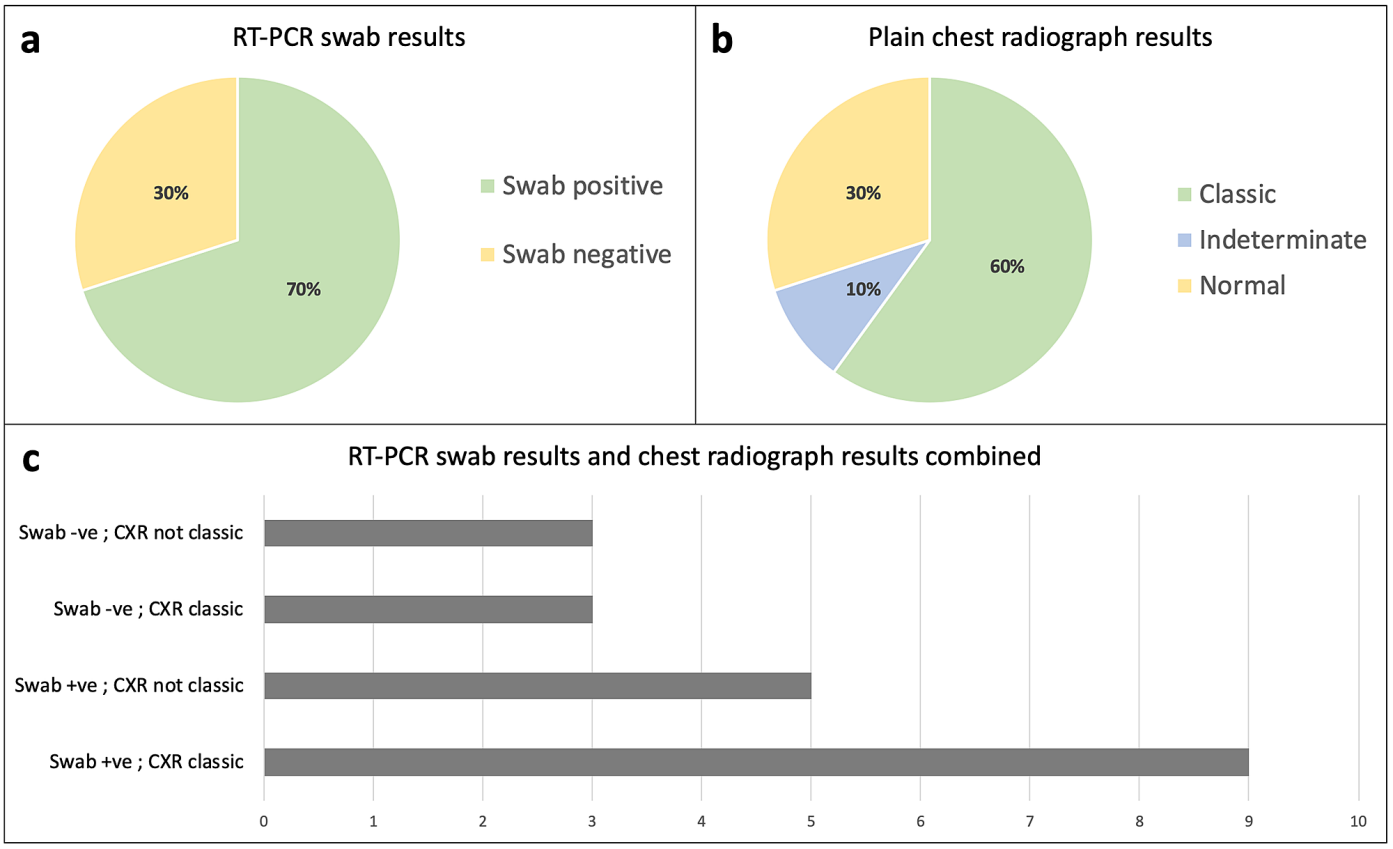
RT-PCR viral swab results and chest radiograph reports Reverse-transcription polymerase chain reaction (RT-PCR) viral swab results (2a) and plain chest radiograph reports (2b) of patients included on this pathway. 2c illustrates merged data for RT-PCR swab results and chest radiograph results (N = 20).

A reverse-transcription polymerase chain reaction (RT-PCR) swab of the upper respiratory tract for SARS-CoV-2 was positive in 14 patients (Figure 1). All patients had a chest radiograph reported by a consultant radiologist or reporting radiographer, of which 12 were ‘classical’ for COVID-19 changes (Figure 1). The decision to move to computed tomography pulmonary angiogram (CTPA) was left to the responsible clinician. A CTPA was performed in four patients and was positive for a pulmonary embolism in one patient.

**Figure 2:**
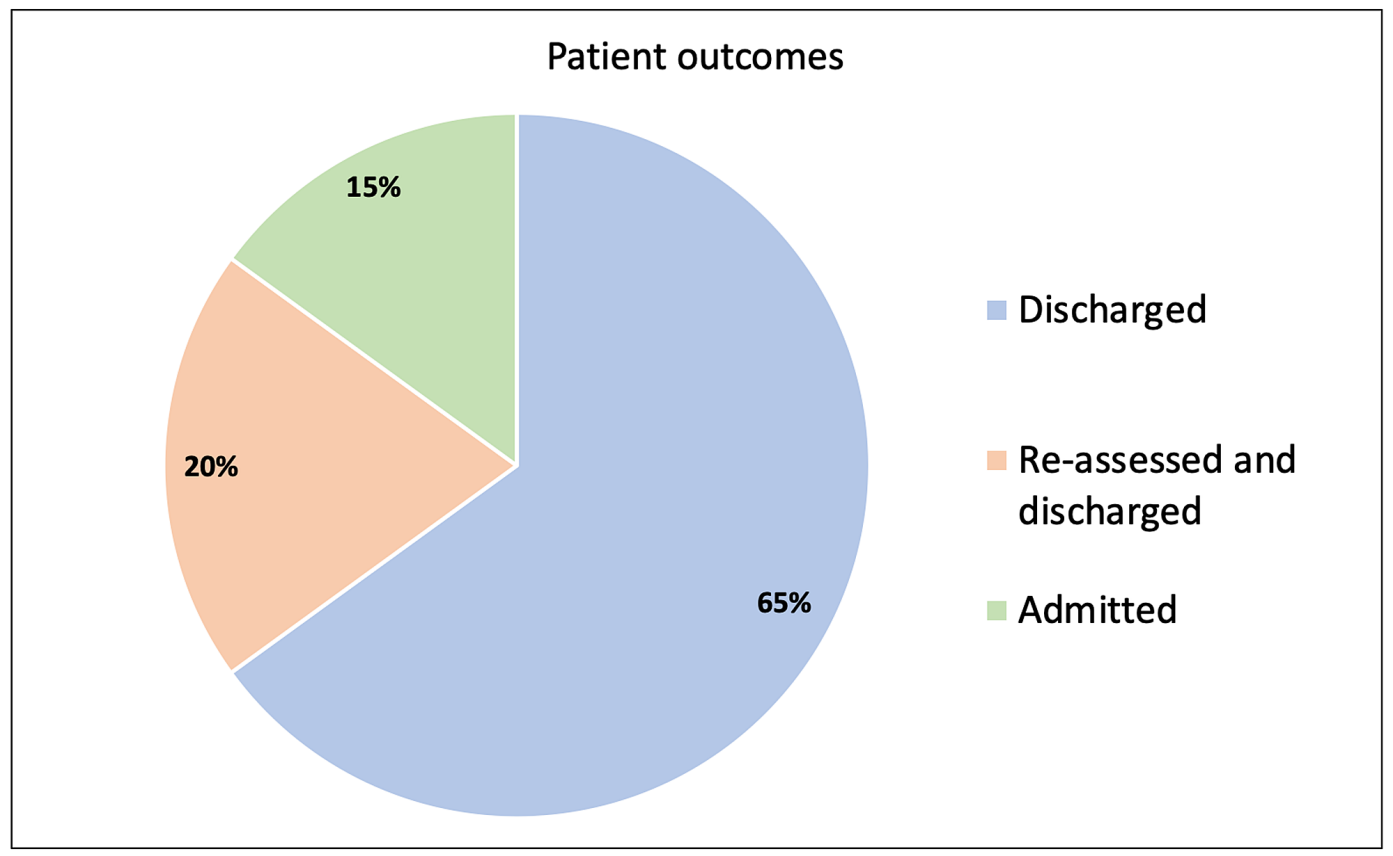
Patient outcomes Patient outcomes after inclusion on the ambulatory care pathway (N = 20).

Patient outcomes are shown in Figure 2. Eighty-five percent of patients successfully avoided hospital admission. Three patients (15%) required an admission following re-assessment: one was admitted for observation, whilst the other two were admitted for oxygen therapy. These three patients avoided hospital admission for a combined total of 10 days. Zero patients died as part of this study. 

**Figure 3:**
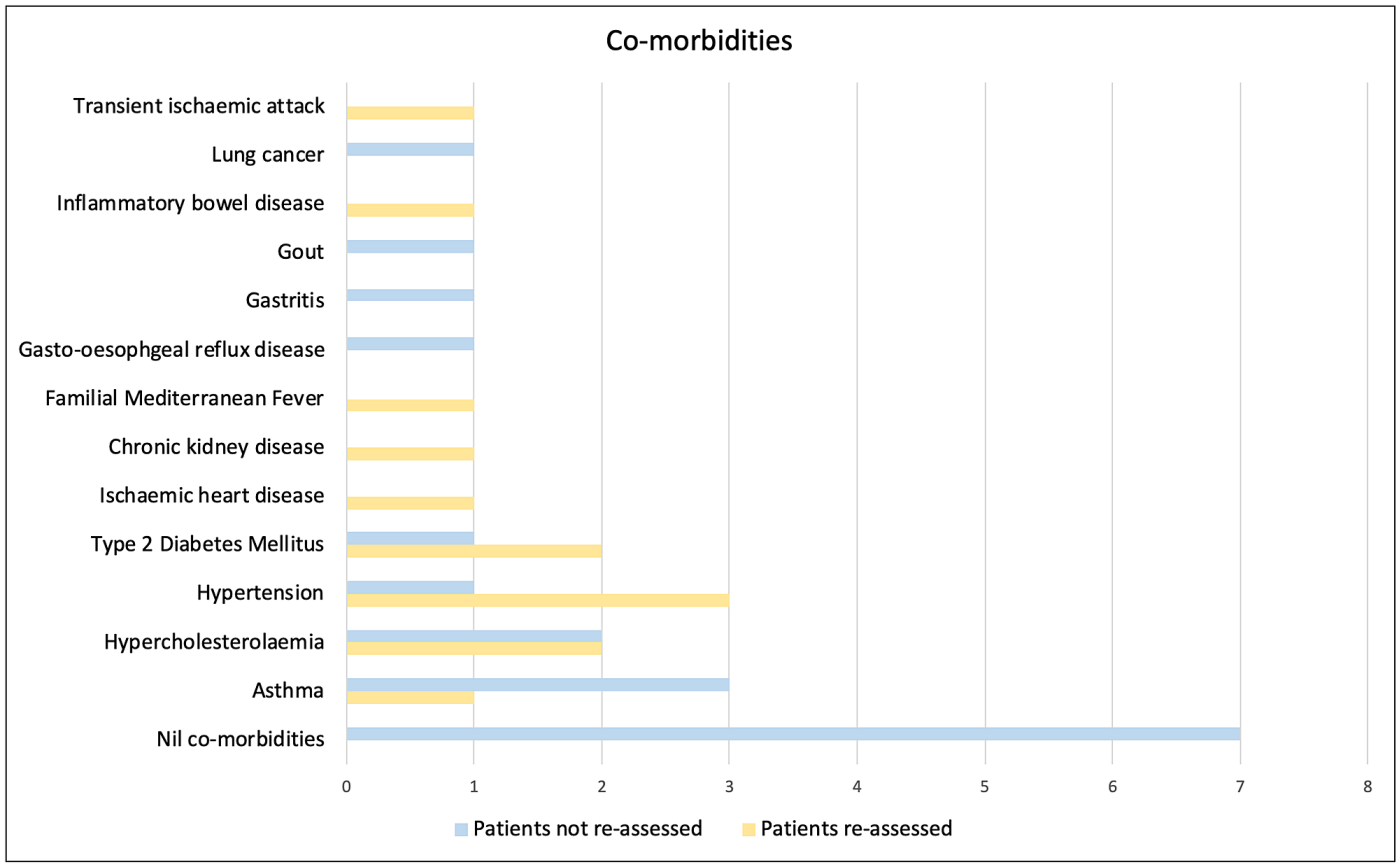
Patient co-morbidities Patient co-morbidities in the cohort who did not require re-assessment compared with the cohort who did require re-assessment (N = 20).

**Figure 4:**
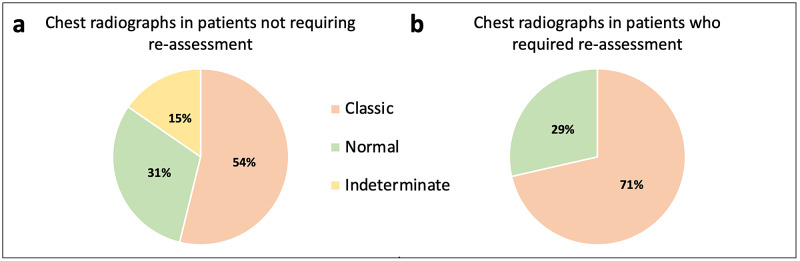
Chest radiograph reports Chest radiograph reports in the cohort who did not require re-assessment (5a; N = 13) compared with the cohort who did require re-assessment (5b; N = 7).

The seven patients who were recalled for face-to-face re-assessment in the ED, of whom five were male and two were female, had a mean age of 57. Those who required admission had a mean age of 65 and were all over the age of 60. All patients with either ‘Asian or Asian British - Indian’ or ‘Asian or Asian British - Pakistani’ ethnicity in the original cohort required re-assessment. Each patient requiring re-assessment had at least one co-morbidity (Figure 3); three patients had two or more co-morbidities. Pulmonary infiltrates on chest radiograph were more common in patients requiring re-assessment in the ED compared to patients not requiring re-assessment, as shown in Figure 4. Three patients who were reassessed had a CTPA: all were negative for pulmonary embolism.

## Discussion

During the COVID-19 pandemic there were growing concerns of the increasing demand for inpatient hospital beds and supplemental oxygen. A decision was made by Trusts across London to adjust target oxygen saturations amongst COVID-19 patients. At the Royal Free London NHS Foundation Trust, COVID-19 patients without underlying respiratory disease had target oxygen saturations of greater than 90%. This target is in line with research demonstrating poorer outcomes associated with oxygen saturations below 90% in COVID-19 pneumonia [4]. Identifying COVID-19 patients with oxygen saturations of 90-94% who could be safely discharged from the ED was difficult due to uncertainties of the trajectory of the disease course and limited follow-up options. As such, an ambulatory care pathway was implemented to aid the discharge of these patients from the ED. Twenty patients were discharged on this pathway between April and May 2020. Sixty-five percent of patients were successfully discharged without requiring any further face-to-face clinical assessment, whilst 85% of the original cohort did not require admission to hospital. 

Six patients on this pathway were recalled for face-to-face re-assessment due to either fluctuating oxygen saturations or worsening dyspnoea. One patient self-presented to the ED based on safety-netting advice, and subsequently required an admission. Literature has shown a correlation between mortality and the clinical feature of dyspnoea in COVID-19 [4], in addition to the importance of pleuritic chest pain as a symptom [5]. This literature, in addition to individual patient accounts, emphasises the importance of telephone follow-up to assess, not just the oxygen saturations, but also the trajectory of symptoms. 

Our results suggest that increasing age and the presence of co-morbidities increase the likelihood of repeat ED assessment and hospitalisation in COVID-19. Cardiovascular co-morbidities were more common in the cohort of patients who required re-assessment, which supports evidence that obesity, hypertension, cardiovascular disease, and diabetes are associated with increased mortality in COVID-19 [6]. This reiterates the importance of clinical acumen in combination with investigations in discharge decision-making, whilst also emphasising the importance of shielding elderly and co-morbid individuals.

Interestingly, all patients with either ‘Asian or Asian British - Indian’ or ‘Asian or Asian British - Pakistani’ ethnicity in the initial cohort required re-assessment. Although this sample size is too small to draw exact conclusions regarding the risk of COVID-19 within different ethnic groups, this finding reflects evidence that the Black, Asian and minority ethnic (BAME) population have an increased risk of deterioration from COVID-19 [7].

Chest radiographs that were ‘classical’ for COVID-19 changes were more common amongst the cohort of patients who required re-assessment in the ED, supporting the significance of radiological findings as a prognostic factor in COVID-19 patients [8]. Despite this, there were patients with ‘classical’ chest radiographs who did not require re-assessment, reiterating the importance of a combination of prognostic markers. 

Inclusion criteria aimed to select patients with a high clinical suspicion of COVID-19, rather than a positive RT-PCR result. This is important due to limitations of RT-PCR testing [9,10], as well as timely decision-making being necessary in the ED. Limitations of RT-PCR testing were reflected by three patients with ‘classical’ COVID-19 chest radiographs having a negative RT-PCR result. Clinical suspicion was defined by typical clinical features, exposure combined with lymphopenia [11], or typical radiological changes [12]. Information provided to clinicians on factors that may influence admission was based on existing literature and included C-reactive protein (CRP) [13], troponin [14], percentage of pulmonary infiltrates on chest radiograph [8], co-morbidities [15], and respiratory rates [16]. Other prognostic markers, such as hyperferritinaemia, may have also been considered prior to discharge. However, a balance was struck between ensuring safe discharge and collecting excessive data that may result in undue anxiety regarding discharge of patients with this novel disease.

Monitoring and follow-up in this study were designed to replicate real-life clinical situations. Three times a day monitoring of SaO2 in the community aimed to mimic inpatient monitoring of a stable patient, whilst limiting laborious monitoring that could negatively impact patient compliance. Night-time oxygen saturations were not monitored due to nocturnal variations in oxygen saturation [17]. Patients were followed-up at 48 hours post-discharge, in line with typical management of unwell ambulant patients at Barnet Hospital.

Patients were taught to use the pulse oximeter by the discharging clinician. Discrepancies in the education provided to patients raise a possible limitation of this pathway. These discrepancies may have existed because training was given to discharging clinicians via email, but also given the context of such conversations in a busy ED. Remote training via email was provided in order to adhere to social distancing regulations within the hospital setting. The information sheet given to patients aimed to combat these discrepancies by offering some instructions for the correct usage of the pulse oximeter.

Although admission practices vary between senior clinicians, it is likely that a considerable proportion of the patients who were not admitted to hospital on this pathway would have otherwise been admitted in the absence of this pathway. As such, the primary objective of this study to utilise home pulse oximetry to safely avoid admissions was successfully met. Given the rapid improvement in oxygen saturations observed in many of the subjects, it is expected that most patients included in this pathway may have required only a short admission had the pathway not existed. Although cost-saving was not the intended outcome of this study, the reduced cost of managing pneumonia in the community helps to make a compelling case for further development of this and similar pathways [18].

The secondary objective, to identify patients in whom further investigations were required, was also met. Telephone follow-up consultations identified a total of seven patients who required a second assessment. Further, this pathway identified a patient who was later found to have an alternative diagnosis. Interestingly, one patient used their pulse oximeter on a relative at home, which prompted attendance to the ED and subsequent admission to the intensive care unit. 

## Conclusions

Ongoing development of the pathway will focus on providing a dynamic service responsive to the needs of the population. This will be important in the event of further waves of cases, as well as in providing additional support during winter pressures. This study, when combined with similar data from other trusts, illustrates the potential for safe and cost-effective ambulation of patients with COVID-19 infection using home pulse oximetry with early remote follow-up. The protocol developed by the authors is useful in identification of patients in whom ambulation is likely to be safe. Future research should focus on the assessment of various prognostic markers and their potential power in determining disease trajectory, and therefore, likelihood of successful discharge.
